# Distribution of HPV Genotype in Invasive Cervical Carcinoma and Cervical Intraepithelial Neoplasia in Zhejiang Province, Southeast China: Establishing the Baseline for Surveillance

**DOI:** 10.3390/ijerph120910794

**Published:** 2015-09-02

**Authors:** Xiao-Xian Xu, Jian-Song Zhou, Shu-Hui Yuan, Hua Yu, Han-Mei Lou

**Affiliations:** 1 The Key Laboratory of Radiation Oncology of Zhejiang Province, Banshan Bridge, Guangji Rd #38, Hangzhou 310022, China; E-Mails: dr.cindyxu@foxmail.com (X.-X.X); zhoujs@zjcc.org.cn (J.-S.Z.); zhuhuaxiang@zju.edu.cn (S.-H.Y.); ayuhua@126.com (H.Y.); 2 Department of Gynecologic Radiation Oncology, Zhejiang Cancer Hospital, Banshan Bridge, Guangji Rd #38, Hangzhou 310022, China; 3 Department of Gynecologic Oncology, Zhejiang Cancer Hospital, Banshan Bridge, Guangji Rd #38, Hangzhou 310022, China

**Keywords:** HPV, vaccine, cervical, ICC, CIN, genotype

## Abstract

Human papillomavirus (HPV) are firmly established as the principal causative agent for cervical carcinoma. Current vaccines may provide some protection for women from cervical carcinoma linked to HPV genotype 16 and 18. This may be the best vaccine for Western women, but the geographical variation in HPV distributions may not make it the most appropriate vaccine for China or Asia. This study provided an observational, retrospective, hospital-based cross-sectional study on the distribution of HPV genotypes among 5410 women with invasive cervical cancer (ICC) or cervical intraepithelial neoplasia (CIN). Overall, the positive rates of the four HPV types included in current prophylactic vaccines were counted, the two high-risk types (HPV-16 and -18) covered by current vaccines represented 66.9% of women with squamous cancer, 55.0% with adenocarcinoma, 64.9% with adenosquamous carcinoma and 77.4% of other type ICC, as well as 59.5% of CIN III, 45.0% of CIN II and 38.1% of CIN I cases. As expected, two low-risk types (HPV-6 and -11) included in the quadrivalent vaccine did not show good coverage data. Particularly worth mentioning is the fact that the addition of HPV-52 and -58 to the vaccine cocktail would increase cancer protection in our population, potentially preventing up to beyond 16% of squamous/adenosquamous carcinoma and other type of cervical cancers, and 7.75% of adenocarcinomas. It might also potentially reduce the rate of CIN III by a further 28.6% and CIN II and I by a third. This study established the baseline for surveillance in Zhejiang Province, and provides data for further vaccine designs: a quadrivalent HPV vaccine covering HPV-16/-58/-18/-52, would be more welcome in our region in the forthcoming year compared to the currently available vaccine.

## 1. Introduction 

Invasive cervical cancer (ICC) ranks as the third most common malignancy and the fourth cause of cancer-related death among women worldwide [1,2], with an estimated 528,000 new cases and 266,000 deaths in 2012 [3]. Although well-organized screening and early therapeutic treatment have dramatically reduced the risk, cervical cancer continues to be a global concern, especially in developing areas, which account for 83% of all cases [4].

HPV is firmly established as the principal causative agent for cervical carcinoma. To date, more than 200 HPV genotypes have been identified, 40 of which are sexually transmitted and 15 of these are carcinogenic and classified as high-risk human papillomaviruses (hr-HPVs) [5,6]. Infection plus subsequent events can lead to HPV genome integration and unregulated expression of the pivotal viral oncogenes, E6 and E7. This promotes malignant transformation and immortalization of cervical host-cells [7]. 

HPV vaccines have been commercially available since 2007. The bivalent vaccine Cervarix^®^ (GlaxoSmithKline Biologicals, Middlesex, UK) targets HPV-16/-18 and the quadrivalent vaccine Gardasil^®^ (Merck, Whitehouse Station, NJ, USA) targets HPV-6/-11/-16/-18. The vaccines may offer some crossover and protection from other HPV types, but vaccinated women will not gain significant protection against oncoviruses other than 16 and 18. 

Therefore, the knowledge of the regional HPV genotype is crucial to guiding estimates of the effectiveness of vaccination programs, health economic calculations and the development of improved vaccines. HPV-16 and -18 are the two most common hr-HPVs types but there are geographic variations. A survey of healthy women by the International Agency for Research on Cancer established that some genotypes were particularly prevalent in different continents (e.g., HPV-45 and -33 in Africa; HPV-33 and -31 in Europe; HPV-31,-33 and -45 in America and HPV-58 and -52 in Asia [5]. Five years later, the Retrospective International Survey and HPV Time Trends Study Group reported the prevalence of HPV subtypes in cervical cancer and confirmed that the geographic variations in HPV distribution in healthy women was reflected by a similar distribution in the cancer biology [5].

The objective of this epidemiological survey is to describe the distribution of HPV genotypes amongst women with ICC and CIN from Zhejiang Province, a coastal region in southeast China. China’s population is unvaccinated and equivalent to 19% of the total world population. This is potentially a massive health market for vaccine manufacturers and it may not be appropriate for China to commission currently available vaccines. The aim of this work was to establish the baseline prevalence of HPV genotype distribution in a large series of consecutive cases of cancer in anticipation of future HPV surveillance and the commission of a second generation, but locally appropriate vaccine. 

## 2. Materials and Methods

### 2.1. Study Population

During August 2008 and June 2013, a total of 5410 woman with an ICC or CIN had exfoliated cervical cell samples examined and reviewed by the pathological quality control center of Zhejiang Province at Zhejiang Cancer Hospital. Of these women, 4250 were histologically diagnosed as ICC, including 3870 (81.9%) squamous cell carcinoma (SCC), 258 adenocarcinoma (ADC), 91 adenosquamous carcinoma (ASC) and 31 other type ICC (1595, 1765, 756 and 134 cases for stage I to IV of ICC, respectively), 1160 were CIN (21 as CIN I, 240 as CIN II and 899 as CIN III). The Local Institutional Ethics Committee approved the study.

### 2.2. HPV Detection and Typing

HPV genotyping by HybriMax used an HPV GenoArray Test Kit (HybriBio Ltd., Chaozhou, China) according to the manufacturer’s instructions [8]. This assay can differentiate 21 HPV types, including 14 hr-HPV types (HPV-16, -18, -31, -33, -35, -39, -45, -51, -52, -56, -58, -59, -66 and -68), five low-risk HPV (lr-HPV) types (HPV-6,-11, -42, -43 and -44) and two unknown-risk types (HPV-53 and -81 equivalent to CP8304). Briefly, PCR was performed in a 25 μL reaction mixture containing 5 μL extracted DNA, 0.75 μL DNA Taq polymerase and 19.25 μL PCR-mix solution containing MY09/11 primer system. The PCR protocol was: denaturing at 95 °C for 9 min, followed by 40 cycles of 20 s at 95 °C, 30 s at 55 °C, and 30 s at 72 °C, at last a finally extension at 72 °C for 5 min. A positive control and a negative control were run in each PCR analysis process to control for possible contamination and accuracy. The kit can simultaneously identify 21 HPV genotypes: adding NBT/BCIP solution to display the results, a positive result was indicated by a clearly visible indigo dot. The HPV-genotype result was determined by the position of the HPV-genotype probes on the microarray chip. Multiple dots indicated multiple infections.

### 2.3. Statistical Analysis

The data were key-entered twice using SPSS 16.0 software package (IBM Corp., Armonk, NY, USA). The χ^2^ test was used to compare HPV prevalence across groups. Statistical significance was determined at *p* < 0.05 level.

## 3. Results 

### 3.1. HPV Positive Rate and Infection Status

4405 (81.4%) of the 5410 cervical samples from women with known pathology had detectable HPV: 80.3% with ICC, compared to 85.5% with CIN (*p* < 0.001). The 4250 samples of ICC, included SCC, ADC, ASC and other carcinomas, the rate of hr-HPV genotype was higher with SCC than ADC (*p* < 0.001), but no difference was observed between SCC and ASC (*p* = 0.08). Of the 1160 samples from women with CIN, the HPV positive rate was higher with CIN III than CIN I (*p* < 0.001) and CIN II (*p* < 0.001), but no difference was seen between CIN I and CIN II (*p* = 0.97) The proportions of single- or multiple-type (co-, tri-, tetrainfection or more) infection are shown in Table 1. Infection with just one genotype seemed to be more likely as the severity of pathology increased. Conversely, multiple-type infection was more likely with lower grade disease.

**Table 1:** Detection of HPV and proportion of multiple-type infection according to cervical pathology status.

Cervical Pathology Status (Total No. Recruited)	HPV Positive Rate (%)	Single-Type Infection (%)	Multiple-Type Infection (%)		
			Co-Infection	Tri-Infection	Tetra- or More Infection
Cervical intraepithelial neoplasia (CIN)					
CIN1 (21)	81.0	47.6	28.6	0	4.8
CIN2 (240)	81.3	55.4	17.5	7.1	1.3
CIN3 (899)	86.8	63.1	18.5	4.7	0.6
Total CIN (1160)	85.5	61.2	18.4	5.1	0.8
Invasive cervical cancer (ICC)					
SCC (3870)	81.9	65.6	13.2	2.8	0.6
ADC (258)	58.1	45.7	9.3	2.7	0.4
ASC (91)	74.7	56.0	14.3	4.4	0
Other type ICC (31)	71.0	41.9	25.8	3.2	0
Total ICC (4250)	80.1	61.9	13.1	28.2	0.6

**Figure 1:**
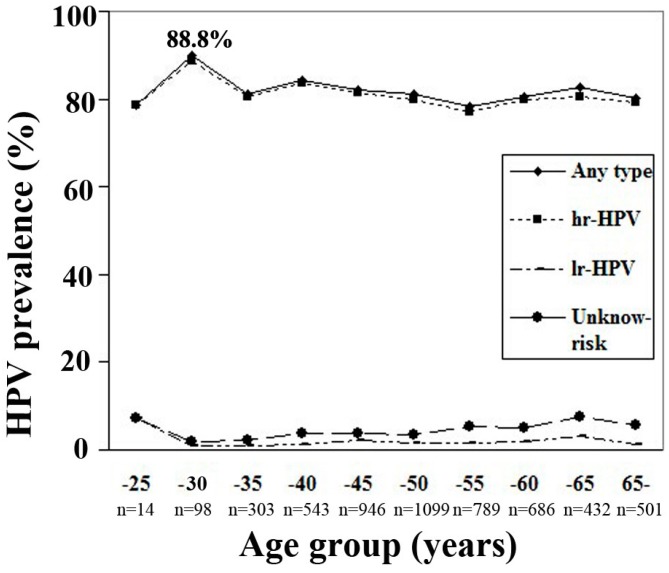
Age-specific of HPV infection in hospital-based female population in Zhejiang Province. High-risk HPV types included HPV-16, -18, -31, -33, -35, -39, -45, -51, -52, -56, -58, -59, -66 and -68; Low-risk HPV types included HPV-6,-11, -42, -43 and -44 and unknown-risk types included HPV-53 and -81(equivalent to CP8304). The peak of hr-HPV infection reached 88.8% in 26–30 years old women.

The mean age of the positively detected women was 49.27 (range 16–93 years). Figure 1 shows the age-specific prevalence for categories of HPV infection. The peak of hr-HPV infection reached 88.8% in 26–30 year old women.

### 3.2. HPV Epidemiological Distribution 

**Figure 2:**
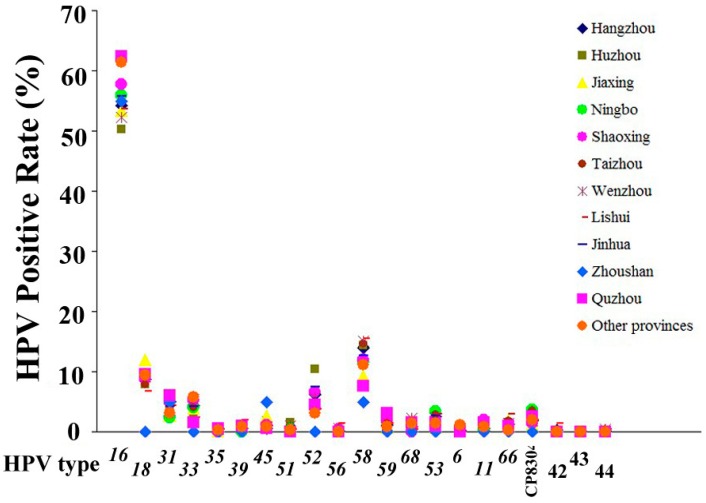
Geographic variation of HPV infection in hospital-based female population in Zhejiang Province. HPV attribution varied substantially among regions in Zhejiang Province, HPV16 predominates the pathogenesis among all regions, followed by HPV58, 18, 52 and other types.

HPV-16, -58, -18 and -52 were the most prevalent types found in Zhejiang Province. Figure 2 shows that their prevalence varied substantially in the regions. Overall, HPV-16 dominates the pathogenesis both in CIN and ICC in all regions.

**Figure 3:**
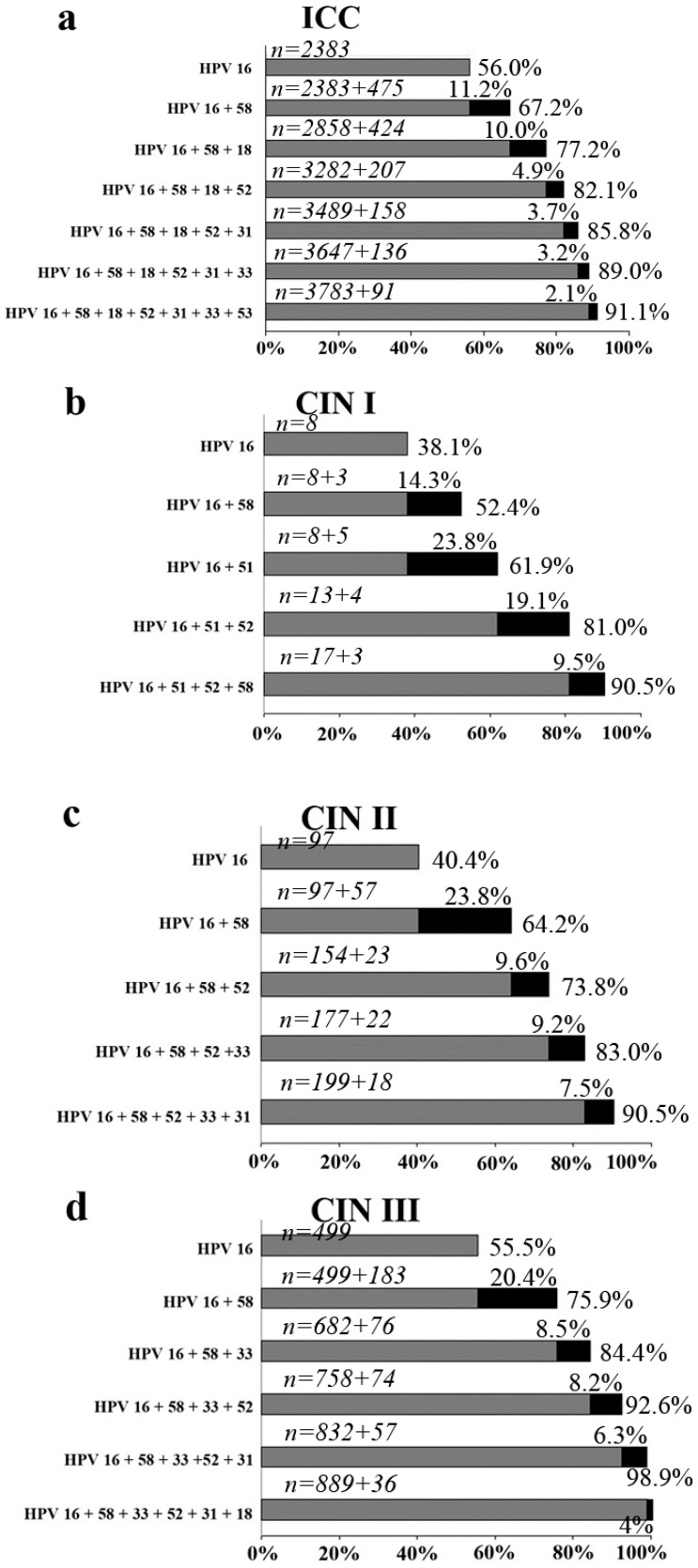
Cumulative attribution rates of HPV types for invasive cervical cancer and cervical intraepithelial neoplasia. (**a**) invasive cervical cancer (**b**) CIN grade I (**c**) CIN grade II (**d**) CIN grade III. Marginal attribution rates conferred by individual HPV types and cumulative attribution rates are listed.

*Invasive cervical cancer.* The relative distribution of HPV types among the 4250 cases of cervical carcinoma, regardless of the infection status (single- or multiple-type or none and pathologic type is shown in Figure 3a. The most prevalent type was HPV-16 (56.1%), followed by HPV-58 and -18 with relatively low rates (11.2% and 10.0%), and then another four HPV types (HPV-52/-31/-33/-53) with lower rates ranging from 4.9% to 2.1%.

*Cervical intraepithelial neoplasia.* The four most prevalent types in CIN I were HPV-16 (38.1%), HPV-51 (23.8%), HPV-52 (19.1%) and HPV-58 (14.3%); in CIN II they were HPV-16 (40.4%), HPV-58 (23.8%), HPV-52 (9.6%) and HPV-33 (9.2%); in CIN III they were HPV-16 (55.5%), HPV-58 (20.4%), HPV-33 (8.5%) and HPV-52 (8.2%). As shown in Figure 3b–d, HPV-16 plus -58 accounted for the majority of CIN, with attribution rates of 52.4%, 64.2% and 75.9%, respectively.

### 3.3. HPV Contribution in ICC 

**Figure 4:**
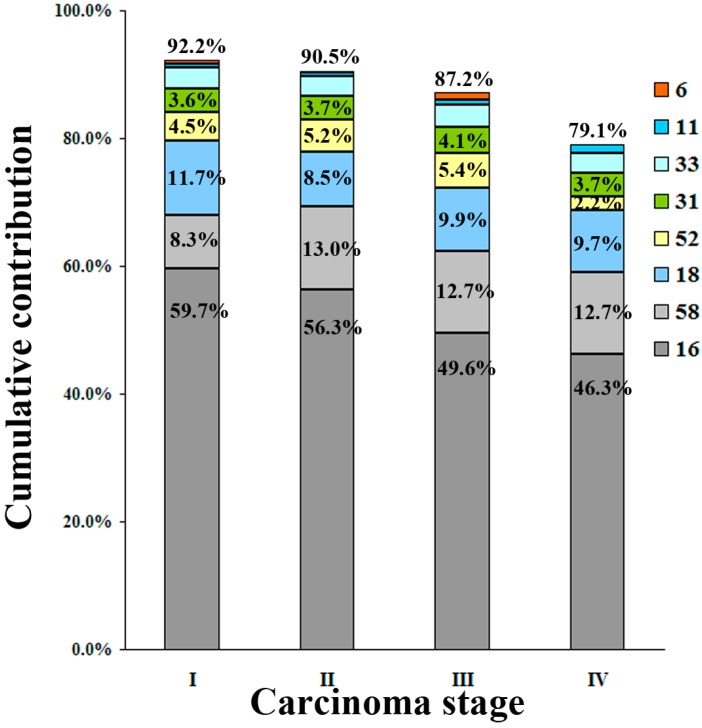
Cumulative contribution of the six most common HPV types for each stage of invasive cervical cancer (ICC). HPV-16 attributed to the majority of ICC, and marginal attribution rates conferred by individual HPV types and cumulative attribution rates are listed.

The cumulative contribution of six hr-HPV and two lr-HPV types for each stage of cancer shows a generally lower detection with advancing stage (Figure 4). 

**Figure 5:**
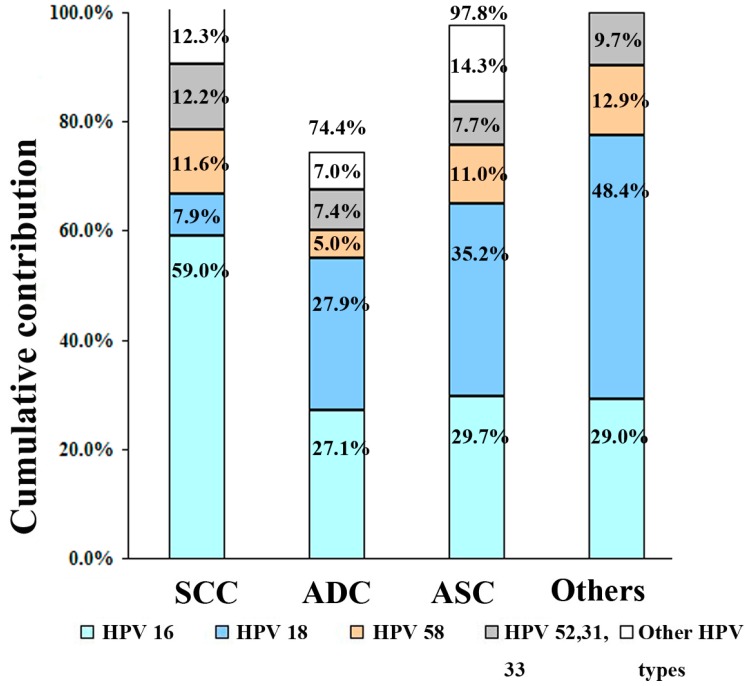
Cumulative contribution of HPV types for pathologic types of invasive cervical cancer. Attribution rates conferred by individual HPV types and cumulative attribution rates are listed.

HPV-16, -58 and -18 were the most prevalent types found in ICC, HPV-16 attributed to the majority of ICC staging (59.7% to I, 56.3% to II, 49.6% to III, and 46.3% to IV), with the subsequent types showing lower attribution rates (HPV-58: 8.3%, 13.0%, 12.7% and 12.7%, respectively; HPV-18: 11.7%, 8.5%, 9.9% and 9.5%, respectively). HPV-16 plus -58 accounted for the majority of ICC stage with attribution rate of 68.0%, 69.3%, 62.3% and 59%, respectively (Figure 4). Stratified by pathology types, HPV-16 accounted for the majority (58.7%) of SCC, with the subsequent types showing much lower attribution rates (HPV-58: 11.5%; HPV-18: 7.9%). In contrast, HPV-18 ranked first for ADC, ASC and other types of ICC (27.9%, 35.2% and 48.4%, respectively), followed closely by HPV-16 (27.1%) in ADC, (29.7%) in ASC and (29.0%) in other type of ICC (Figure 5).

### 3.4. Vaccine Coverage

The positive rates of the four HPV types included in current available prophylactic vaccines were counted. The two high-risk types (HPV-16 and -18) covered by current available vaccines represented 66.9% of SCC, 55.0% of ADC, 64.9% of ASC, 77.4% of other type ICC, 59.5% of CIN III, 45.0% of CIN II and 38.1% of CIN I cases. The two low-risk types (HPV-6 and -11) included in the quadrivalent vaccine only represented a small proportion of ICC (1.2% of SCC and 1.3% of ADC) and CIN (1.9% of CIN III, 2.5% of CIN II and none of CIN I). Particularly worth mentioning is that addition of HPV-52 and -58 to the vaccine cocktail would result in the biggest marginal increase in coverage, with 16.6% for SCC, 7.8% for ADC, 16.5% for ASC, 16.1% for other type ICC. It might also potentially reduce the rate of CIN III by a further 28.6% and CIN II and I by a third. 

## 4. Discussion

This study adds to the growing literature on the distribution of HPV genotypes among cervical cancer and its precursors. Controlling HPV infection is thought to be the key to minimizing the impact of cervical cancer. The knowledge of HPV prevalence, its subtype and age distribution might guide local public health policies and prevention measures, including vaccination [9]. Hence, it is important to conduct studies that characterize HPV infections among specific populations, and our data were much closer to the carcinogenesis of HPV in the disease state, compared to the general population.

There are several limitations of the data in this report. We do not know how many women had cancer from our region but never had a diagnosis, and this could introduce as certain bias. Another source of potential bias is false negative laboratory reporting. In order to provide internationally comparable data, High-Risk Hybrid Capture 2 (HR HC2) was selected. In our study, the HPV positive rate was only 80.3% of women with ICC, yet others have found values of up to 99.7% [10]. Part of the reason was that the HPV was associated with only 58.1% of ADC. This may be partly due to geographic variation since the data suggests a significant local variation in the prevalence of different HPV genotypes. In part it might be attributable to intrinsic viral characteristics, lower prevalence of lr-HPV than of other oncogenic types, or a lack of adequate detection with existing HPV assays. There was no doubt that HPV-16 has overall importance in both CIN and ICC. The relatively high contribution of HPV-52 and HPV-58 to SCC in East Asia has been reported previously [11]. The current study further confirmed this fact and provided additional information. We showed that HPV-58 and HPV-52 ranked in second and fourth position as the genotypes associated with invasive cancer (second and fourth in SCC, third and fifth in ADC, third and fourth in ASC and third and fifth in other type ICC in each histological group), and always ranked within the top four among CIN lesions. Of note, HPV-18 was less common in CIN I, ranked seventh in CIN II, sixth in CIN III and third in ICC (third in SCC and second in the remaining histological groups). This underrepresentation in precancerous lesions was in accordance with previous studies [12], suggested its increase is vital to tumor progression. Furthermore, our findings confirmed that the detection of lr-HPV types, mainly HPV-6 and -11 in both CIN and ICC is a rare oncological event.

Multiple HPV infections occurred in 24.3% of women with CIN (32.4% for CIN I, 25.9% for CIN II and 23.8% for CIN III) and 16.5% of ICC (16.6% for SCC, 12.4% for ADC, 18.7% for ASC and 29% for other type ICC). So far, neither the role played by each HPV type found in multiple-type infections in a lesion (in a random fashion or a competitive or cooperative relationship exists), nor the type-specific contribution of HPV to CIN and ICC progression is clear. Interestingly, Jaisamrarn found a paradoxical result that co-infection could increase the risk of progression to a CIN but also increase the chance of clearance [13]. Pista, studying Portuguese women, found a significant association between multiple infections and disease severity [14]. Balbi *et al.* concluded that the infection with multiple HPV types is a significant risk factor for high-grade CIN [15]. The classic study from Trottier showed that infections with multiple HPV may synergistically boost carcinogenesis [16]. However, Cuschieri showed that multiple hr-HPV infection did not have a higher risk than a single hr-HPV infection [17]. In addition, previous studies showed that HPV infection is a “necessary” but “insufficient” factor to cause ICC [9]. Unfortunately, the reasons for this fact are still unknown. Moreover, a surprising finding was that a substantial proportion of cases that were HPV negative, particularly among women with adenocarcinoma (HPV positive rate was only 58.1%), by contrast with the expectation of HPV as a recognised universal cause of cervical cancer, suggesting that, in addition to HPV, other potential factors also play an important role in cervical carcinogenesis (particularly in ADC). Hence, further efforts are needed to ascertain which conditions, with or without these HPV types, could induce ICC.

The five most common hr-HPV types in the general worldwide female population are HPV-16, -18, -31, -58 and -52, while the rank varies by geographical regions. HPV-16/-58/-18/-52 were the most commonly identified types in our population. The attribution of HPV types in southeast China seems different from elsewhere. As HPV immunity is type-specific, this should be considered in prioritizing HPV types for vaccine development. Since 2007, specific vaccination programs have been implemented into clinical practice [18], and an effective HPV vaccination program could decrease the incidence of cervical pre-invasive neoplasia by 51.5% to 66.6%, based on the literature review [19]. The two low-risk types (HPV-6/-11) included in the quadrivalent vaccine did not, as expected, have good coverage data, with a small contribution in ICC (1.1%, 0.9%, 2.0% and 1.5% of each stage, respectively.) and CIN (1.9% of CIN III, 2.5% of CIN II and none of CIN I). Incorporating HPV-52 and -58 in future vaccines would provide the highest marginal increase in coverage for our population from 66% to 82% (difference 16%; 95%CI; 15.0–17.1, not counting cross-protection patterns) and reducing the potential future colposcopy burden by up to a third.

## 5. Conclusions

Our data showed a low prevalence of HPV vaccine types (HPV-6 and -11) and relatively high prevalence of HPV-58 and -52, which could serve as a baseline for future monitoring. Hence, we suggested that in Asia, a quadrivalent HPV vaccine covering HPV-16/-58/-18/-52, would be more useful in the forthcoming years.

## Abbreviations

**HPV**	Human papillomavirus		**SCC**	squamous cell carcinoma
**hr-HPV**	high-risk human papillomavirus		**ADC**	adenocarcinoma
**ICC**	invasive cervical carcinoma		**ASC**	adenosquamous carcinoma
**CIN**	cervical intraepithelial neoplasia			
